# Surgical repair of a residual lesion of mixed-type total anomalous pulmonary venous connection using a vertical vein as a free graft: a case report

**DOI:** 10.1186/s44215-023-00107-5

**Published:** 2023-08-25

**Authors:** Yoshinobu Watabe, Nobuyasu Kato, Haruki Niwano, Yasushige Shingu, Tomonori Ooka, Hiroki Kato, Shinji Abe, Satoru Wakasa

**Affiliations:** Department of Cardiovascular Surgery, Hokkaido University Graduate School of Medicine, Kita-15, Nishi-7, Kita-Ku, Sapporo, 060-8638 Japan

**Keywords:** Congenital heart disease, Mixed-type total anomalous pulmonary venous connection, Left superior pulmonary vein, Vertical vein

## Abstract

**Background:**

Mixed-type total anomalous pulmonary venous connection is a rare condition in which the pulmonary veins possess multiple drainage sites. In the treatment of multiple lesions, it is sometimes necessary to treat only the main lesion first and repair additional residual lesions during follow-up.

**Case presentation:**

We report a case of additional repair of a remaining type Ia lesion in a 19-year-old woman with mixed-type total anomalous pulmonary venous connection who had previously been treated for a type IIa lesion alone. The vertical vein was used as a free graft in the surgery, and the reconstruction was successful without turbulence or kinking.

**Conclusions:**

Our results suggest that the use of the vertical vein as a free graft for the repair of residual type Ia lesions in adulthood allows reliable reconstruction regardless of the spatial relationship between the left atrial appendage and the left superior pulmonary vein.

## Background

Mixed-type total anomalous pulmonary venous connection (TAPVC) is a rare condition in which the pulmonary veins possess multiple drainage sites [1]. In the management of mixed-type TAPVC, such as type IIa + Ia, the major lesions (IIa) are initially treated alone and the repair of the remaining Ia lesion is performed during the follow-up period [2]. However, in some cases, a direct anastomosis between the left atrial appendage (LAA) and left superior pulmonary vein (LSPV) cannot be adopted in the second repair because of the spatial relationship between the LAA, LSPV, and pulmonary artery (PA) trunk. Here, we present a rare case of surgical repair of a residual Ia lesion using a vertical vein (VV) as a free graft.

## Case presentation

**Fig. 1:**
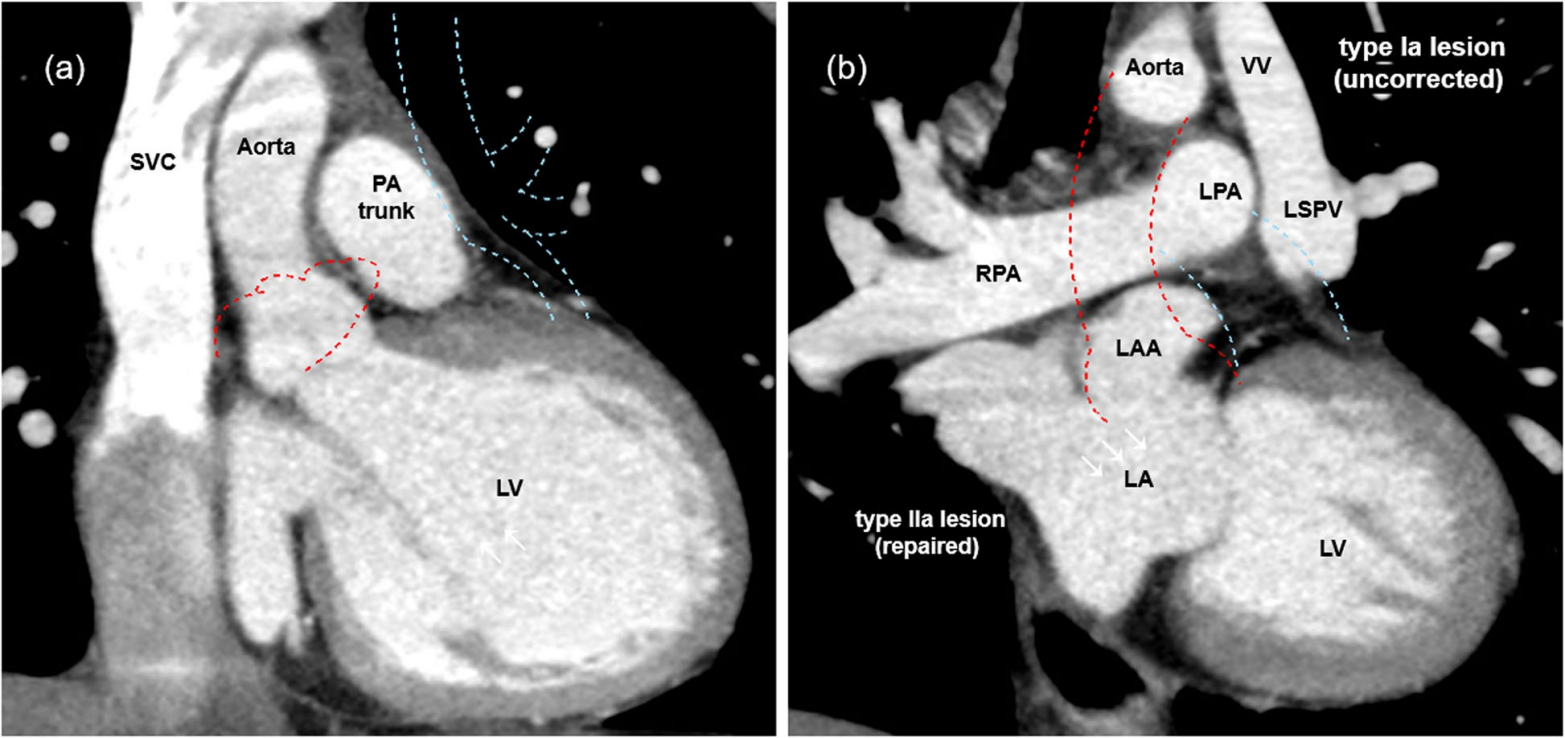
Preoperative computed tomography. **a**, **b** Coronal section of the preoperative computed tomography image reveals that the LAA (red dotted line in (**a**)) is located behind the aorta (red dotted line in (**b**)) and the LAA and LSPV (blue dotted line in (**a**)) are spatially separated by the PA trunk (blue dotted line in (**b**)). LA, left atrium; LAA, left atrial appendage; LPA, left pulmonary artery; LSPV, left superior pulmonary vein; LV, left ventricle; PA, pulmonary artery; RPA, right pulmonary artery; SVC, superior vena cava; VV, vertical vein

We report the case of a 19-year-old woman who was diagnosed with mixed-type TAPVC (IIa + Ia) at 4 months of age. The LSPV was connected to the innominate vein through the VV (Ia), whereas the others returned to the right atrium (IIa). At 4 months of age, surgical repair of the IIa lesion alone was performed, leaving the Ia lesion uncorrected. Residual lesions and hemodynamics were followed regularly after the initial surgery, and reoperation was not considered through childhood because the Qp/Qs value was under 1.5. At the age of 19, the Qp/Qs value increased to 1.7, and magnetic resonance imaging (MRI) showed right ventricular volume overload (right ventricular end-diastolic volume index [RVEDVI], 134.7 ml/m^2^). To prevent right heart failure, a second surgery was planned for the residual Ia lesion. Preoperative computed tomography (CT) revealed that there was a long distance between the LAA and LSPV because the LAA was located behind the aorta and was further right than usual (Fig. 1a). There was also limited space for reconstruction because the LAA and LSPV were separated by the PA trunk (Fig. 1b). These conditions made the reconstruction of the residual Ia lesion using direct anastomosis unfeasible because of the likelihood of kinking or compression of the repair site. Therefore, interposing the LAA and LSPV using the VV as a free graft was considered.

**Fig. 2:**
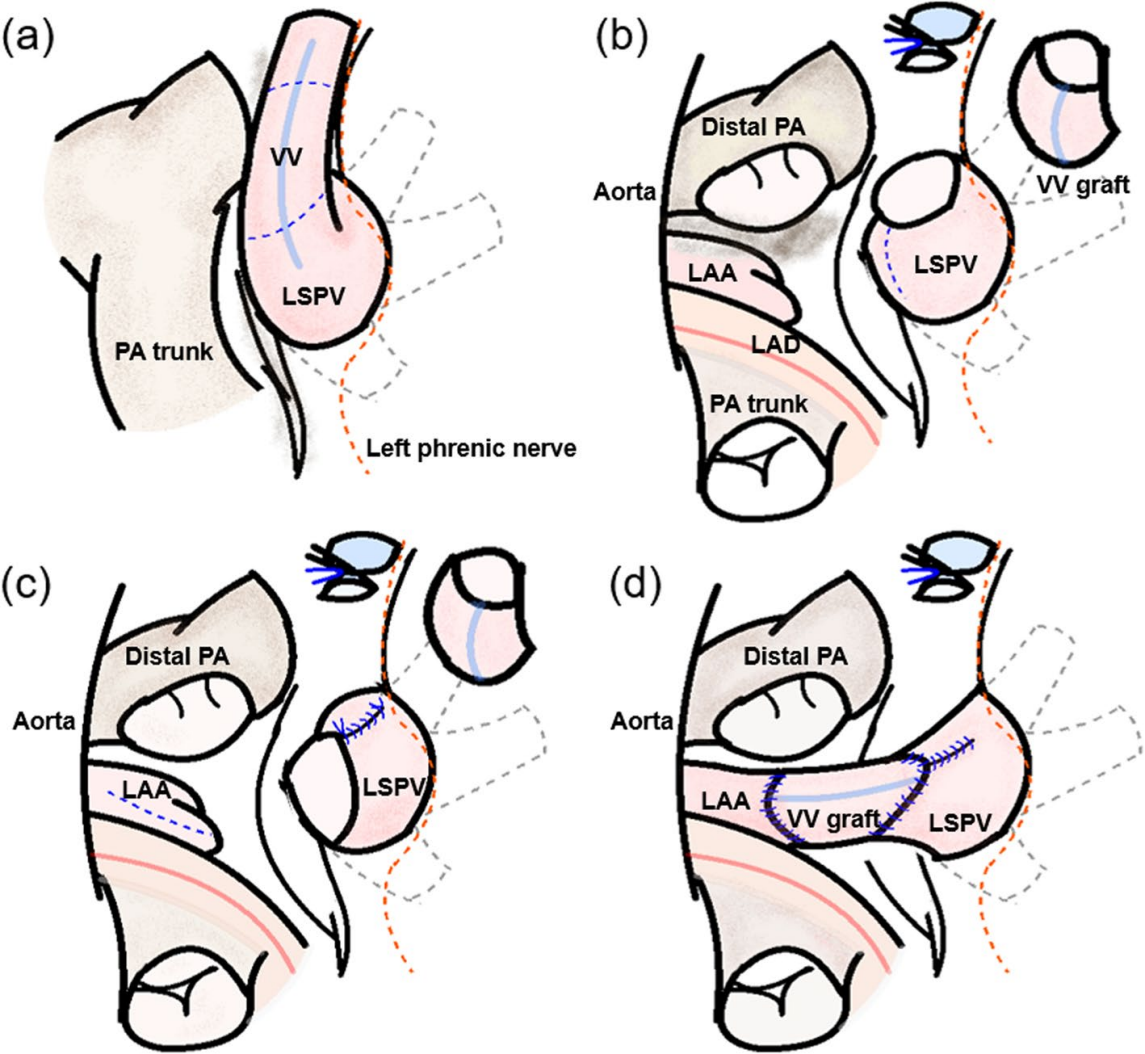
Operative schema. **a** The vertical vein (VV) is exposed and marked. **b** The pulmonary artery (PA) trunk is transected and the VV is harvested as a free graft. **c** An anastomosis site on the left superior pulmonary vein (LSPV) is made by extending the incision ventrally and partially closing the initial opening incision. **d** The VV is interposed between the left atrial appendage (LAA) and LSPV. LAA, left atrial appendage; LAD, left anterior descending artery; LSPV, left superior pulmonary vein; PA, pulmonary artery; VV, vertical vein

**Fig. 3:**
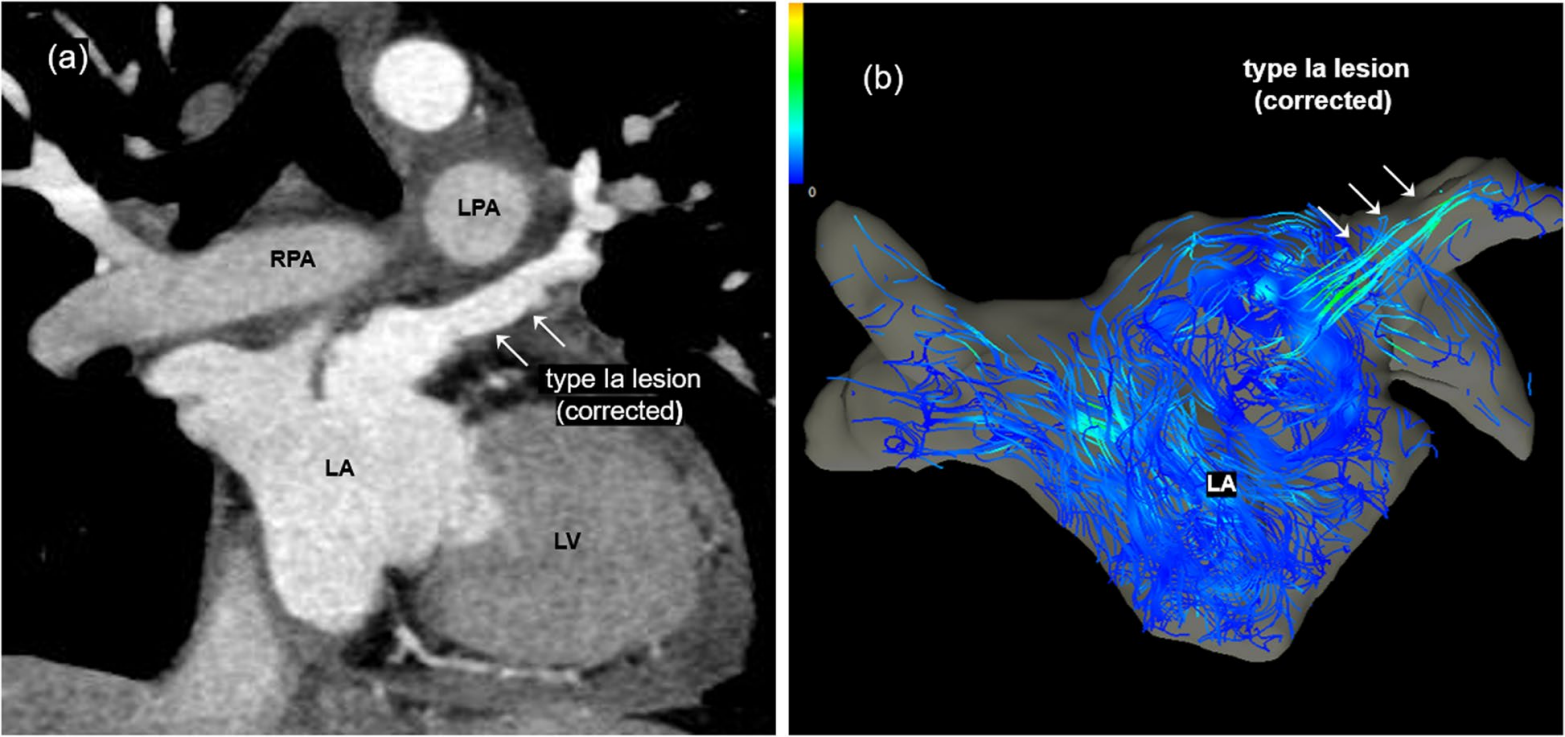
Postoperative computed tomography and 4D flow magnetic resonance imaging. **a** Coronal section of the postoperative computed tomography image demonstrates the repaired type Ia lesion without narrowing and kinking. **b** Postoperative 4D flow magnetic resonance imaging reveals a laminar flow through the corrected type Ia lesion. LA, left atrium; LPA, left pulmonary artery; LV, left ventricle

The operation was performed via median resternotomy, and a cardiopulmonary bypass was established. The PA trunk was transected to expose the LAA (Fig. 2b). Subsequently, the VV was ligated and harvested as a free graft. After resection of the LSPV, the proximal opening was partially closed cranially using a 6–0 polypropylene continuous suture and incised caudally to create an anastomosis site toward the LAA (Fig. 2c). After aortic cross-clamping, the free VV graft was anastomosed to the LAA and LSPV using a 6–0 polypropylene continuous suture to prevent kinking and stenosis of the VV graft (Fig. 2d). After de-clamping, the PA trunk was reattached using direct anastomosis. Blood transfusions were not required. Postoperative examination revealed that there was no narrowing or kinking on CT (Fig. 3a) or intraluminal laminar flow on 4D flow MRI (Fig. 3b). Cardiac MRI showed postoperative decreases in Qp/Qs (1.7 to 1.0) and RVEDVI (134.7 ml/m^2^ to 90.6 ml/m^2^). The postoperative course was uneventful, and the patient was discharged 10 days post-surgery.

## Discussion and conclusions

While treating IIa + Ia TAPVC, the Ia lesion is generally left uncorrected in the initial surgery because direct anastomosis of the vein, which is the approach commonly used [3], is associated with a high risk of stenosis [2, 4]. After the initial surgery, additional repair may be required during the follow-up period. Kimura reported 2 of 3 cases in which the IIa + Ia lesion required reoperation for the residual Ia lesion after partial repair [5]. In residual Ia lesion repair, some studies have reported a direct anastomosis between the LSPV and LAA or using an in situ VV graft [3]. However, these methods may not be feasible in adulthood depending on the spatial relationship between the LAA and LSPV. This is particularly true when the LAA is located behind the aorta, in an unusual placement, or if the LAA and the LSPV are spatially separated by the PA trunk. Another option is required to avoid compression or kinking. The use of autologous pericardium or the PA wall was also considered. Nevertheless, this was a reoperation case, and there was a concern that adequate pericardium for reconstruction could not be harvested. Additionally, the use of the PA wall would require reconstruction of the PA, resulting in a more complicated surgery. Although a prosthetic vascular graft could become a simply useful option, graft occlusion due to thrombosis remains a concern. Therefore, it was determined that the use of the free VV graft was a simple and optimal choice. If the posterior anastomosis or the VV graft was compressed, we considered to cover and protect the VV graft with a ringed-prosthetic vascular graft, or to extend the PA trunk with some kind of compensating materials.

Our report indicates that using the VV as a free graft, regardless of the spatial relationship between the LAA and LSPV, is a viable option. This procedure requires consideration of additional anastomosis, adjustment of the graft length, and possible compression by the surrounding tissues. However, we propose that this procedure is superior to a prosthetic vascular graft and an in situ VV graft in terms of reliable reconstruction. In conclusion, the residual Ia lesion in the TAPVC was successfully repaired using a free VV graft.

## Data Availability

Not applicable.

## Abbreviations

CT: Computed tomography
LAA: Left atrial appendage
LSPV: Left superior pulmonary vein
MRI: Magnetic resonance imaging
PA: Pulmonary artery
RVEDVI: Right ventricular end-diastolic volume index
TAPVC: Total anomalous pulmonary venous connection
VV: Vertical vein
